# Cystic Lung Disease in Down Syndrome: A Case Report and Literature Review

**DOI:** 10.1155/2016/4048501

**Published:** 2016-10-03

**Authors:** Mathew George, John Amodio, Haesoon Lee

**Affiliations:** SUNY Downstate Medical Center, Department of Pediatrics, 450 Clarkson Avenue, Brooklyn, NY 11203, USA

## Abstract

Subpleural lung cysts (SPC) are seen in children with Down syndrome (DS). The incidence and the long term course of these lesions are not known. It is important for pediatricians and pediatric radiologists to be aware of these lung lesions since the DS patients' longevity has increased and they have greater frequency to encounter the clinicians. Autopsy and the radiology series have shown that these lesions are often found in association with congenital heart disease, particularly the endocardial cushion defect and prematurity.

## 1. Introduction

SPC are small cystic dilatations along the subpleural surface of the lungs. On histologic examinations, they were found to communicate with the subpleural alveoli. The cystic lesions in DS were first reported in 1986 in two infants at autopsy [1]. These lesions were associated with congenital heart disease and were often located in the anteromedial region of lungs. We describe an infant with DS who developed viral respiratory illness and was incidentally found to have SPC in the CT scan of the chest.

## 2. The Case

**Figure 1:**
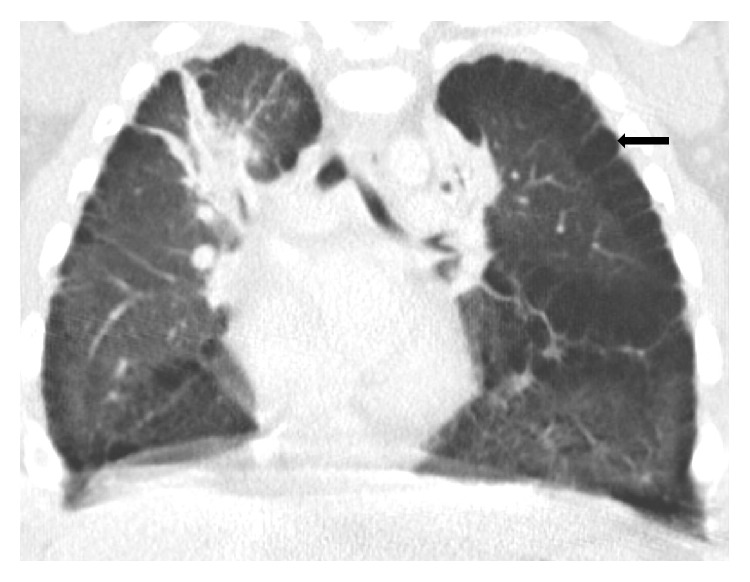
Coronal view of the chest CT shows diffuse subpleural cysts (block arrow) of both lungs along the chest wall and the major fissure of lungs bilaterally.

**Figure 2:**
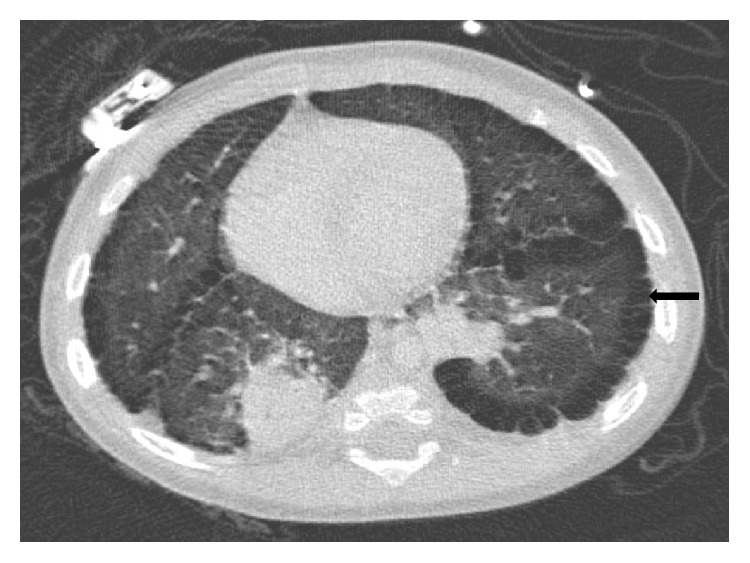
Axial view of chest CT shows the subpleural cysts (block arrow) of both lungs along the chest wall and the major fissure.

A 10-month-old girl of 29-week gestational age and a history of DS with trisomy 21 and chronic lung disease of prematurity was transferred from a community hospital to our Pediatric Intensive Care Unit (PICU) for an otolaryngologic evaluation for stridor. The baby presented to the community hospital 10 days earlier with a history of cough for 4 days, runny nose, tactile fever, and increased work of breathing. She was found to have a temperature of 102°F, respiratory rate of 60 per minute, wheezing, and oxygen saturation of 88% on room air. On chest radiograph (CXR) she was found to have consolidation of left upper and right lower lobes, which were thought to be pneumonia. She was treated with three rounds of nebulized albuterol and ipratropium bromide combination, intravenous corticosteroids, and ceftriaxone. After a consultation with an infectious disease specialist, clindamycin was added to ceftriaxone for the concern for aspiration pneumonia. Blood and viral cultures were negative. When she developed stridor on the 3rd hospital day, she was started on nebulized racemic epinephrine and the supplemental oxygen was increased from 1 liter to 2 liters per minute. She was then transferred to our hospital for ENT evaluation and management of the persistent stridor. A CXR at this time was reported to show an opacity in the left superior mediastinum, which may represent obliquely oriented aorta secondary to the patient's rotation, a right retrocardiac opacity, and hyperinflation. A chest CT scan with intravenous contrast to evaluate an opacity in the left superior mediastinum revealed diffuse peripheral, paraseptal, and subpleural cysts which tracked along the fissure, maximal size of 9.5 mm, predominantly in the upper lobes but throughout both lungs, left side greater than the right and bilateral narrowing of the peripheral bronchi with multiple areas of patchy atelectasis/consolidation (Figures 1 and 2). These features were consistent with SPC seen in DS patients. The ENT evaluation by flexible laryngoscopy did not show a laryngomalacia or vocal cord dysfunction.

The child was treated with nebulized albuterol and normal saline. The antibiotics were continued for 14 days and her condition improved. She was discharged to home to be followed in pediatric pulmonary clinic 2 weeks later with follow-up CXR at that time but the patient did not keep the appointment.

## 3. Discussion

The children with DS have unique problems the clinicians should be aware of. DS, majority caused by trisomy 21 but less frequently translocation and mosaicism, is the most common chromosomal abnormality, affecting one in every 600 to 800 live births. Although the cardiac management is often the dominant clinical focus in DS patients during the neonatal period, the respiratory problems are the most common cause of their hospitalizations and the leading cause of mortality. In a cohort of infants with DS observed from the neonatal period through age 2 years, lung or airway disease accounted for 42% of hospitalizations. The common respiratory problems were their predisposition to frequent respiratory infections, sleep disordered breathing, laryngomalacia, tracheobronchomalacia, tracheal bronchus, subglottic stenosis, pulmonary hypertension, and subpleural cysts [1].

There are only a few reports of SPC in DS in the literature. SPC are small cystic dilatations along the subpleural surface of the lungs. The association of these cysts with DS was first reported in 1986 in two infants at autopsy [2]. The incidence of SPC in DS varies depending on the clinical context of analyzed cases. The prevalence of SPC was reported in a retrospective radiological and autopsy series [3, 4]. Biko et al. published a retrospective review of the CT examinations of 25 children with DS to determine the presence, the location, and distribution of lung cysts and the associated abnormalities, age from 3 months to 20 years. Nine of 25 children (36%) had SPC [3]. Gonzalez et al. reviewed autopsy data on 98 DS infants, 89 live born, age from 3.5 weeks to 12 years, and 9 fetuses or still born. SPC were identified in 18 of 89 (20%) live born infants but none in 9 fetuses or still born infants. In their review of 8000 pediatric autopsy database of non-DS patients, only 2 had findings similar to SPC [4]. Of the total 20 (18 DS patients and 2 non-DS patients) autopsy findings with SPC cases mentioned above, 19 were 2 months or older (10 were older than 1 year), and one was less than 1 month. The first 2 autopsy cases of SPC reported by Joshi et al. were 13 months and 12 months, respectively, but the CXR findings of cystic lesions were present at 7 months and 9 months, respectively [2].

Congenital heart disease was reported as a major risk factor of SPC in DS patients. Gonzalez et al. in their pediatric autopsy series of 98 patients with DS reported that the lung cysts were more frequently associated with congenital heart disease. Lung cysts were present in 20% of live born DS patients with congenital heart disease compared to 4.3% of live born DS patients without congenital heart disease [4]. The most frequent congenital heart lesion described was endocardial cushion defect. However, Biko et al., in their CT case series of 25 children with DS, found SPC in 9 (36%) but did not find an association of SPC with congenital heart disease. Four of the nine SPC were located only in the anteromedial region of the lung, while five others in other parts' of lungs in addition to the anteromedial region [3].

Although the etiology of these cysts is not known, pulmonary hypoplasia is the likely cause [5]. Children with DS were found to have hypoplastic lungs. There were diminished number of alveoli and alveolar ducts, smaller alveolar surface area resulting in enlarged alveoli and alveolar ducts. The subpleural location may be explained by the fact that the peripheral regions of the lung are the region of most recent alveolar formation [4]. The absence of such cysts in fetuses or still born infants with DS supports this hypothesis. The lung hypoplasia was of equal severity whether the child had congenital heart disease or not. Another possible cause of SPC proposed by Gonzalez et al., is ischemia of lung parenchyma from pulmonary arterial occlusion by thrombi or thromboemboli followed by absorption of necrotic tissues resulting in cyst formation. But this etiology is less likely when considering the fact that SPC is absent in BPD where pulmonary parenchymal ischemia is more likely to occur [4, 6].

The size of the SPC reported in literature is 1 to 4 mm in diameter. The lining consists of variable portions of flat and cuboidal epithelial cells. Fibrous connective tissue, lymphatics, and blood vessels are seen between adjacent cysts; the blood vessels and lymphatics are seen in continuity with pleura. The cysts communicate with more proximal air spaces [4]. It is also reported that histologically nonspecific interstitial pneumonitis may be seen as well as paucity of elastin tissue in the walls of cyst [4].

It is generally accepted that the subpleural cyst is best detected by chest CT in children with DS. They are seldom seen in plain CXR. In order to determine the frequency of cystic changes observed on CXR of children with DS, Gyves-Ray et al. reviewed CXR of 45 randomly selected children with DS, age from 1 day to 58 months. Twenty-one had associated congenital heart disease (CHD). They found only one patient that, a 3-month-old boy without a history of CHD, had radiographic findings suggestive of cystic lung disease [7]. Without a CT scan, most cases of SPC would likely have been missed on CXR.

The differential diagnosis of cystic lung disease in this age group includes BPD, Wilson Mikity syndrome (WMS), and CPAM. WMS refers to the chronic lung disease in premature infants, characterized by early development of cystic interstitial emphysema. WMS is now sometimes considered as a part of the spectrum of bronchopulmonary dysplasia [8]. CT scan features of BPD include a mosaic lung parenchymal pattern with areas of low attenuation and focal air trapping, bronchial wall thickening, small subpleural triangular/linear opacities, and parenchymal cyst. Even though our patient had BPD, the CT finding did not show the features of BPD [9–11]. The cyst-like lung lesions in BPD, as reported by Adams et al. in their MRI study, are related to the severity of BPD, and the severity of BPD is related to the low birth weight and the duration of mechanical ventilation. Cyst-like lesions in severe BPD appeared bilaterally, more in the dorsal lung aspect [10]. CPAM is a diagnostic possibility in any cystic lung lesions. However, the cysts are generally localized to one lobe and seldom seen in subpleural area [12]. Congenital pulmonary lymphangiectasia (PL) is a rare developmental disorder involving the lung and is characterized by pulmonary subpleural, interlobar, perivascular, and peribronchial lymphatic dilatation. Autopsy studies suggest that approximately 0.5–1% of infants who are stillborn or die in the neonatal period have pulmonary lymphangiectasia. Diagnosis of PL can be difficult, as many of the respiratory symptoms and radiologic findings are nonspecific. Even though findings in high resolution CT of PL are commonly described as intralobular and peribronchial thickening, patchy ground-glass opacification, pleural effusion, and pleural thickening, the PL can occasionally present as cystic lung lesion [13, 14].

Although the clinical relevance of SPC in Down syndrome is poorly understood, it is still important not to confuse this common finding with other pathologies and cause unnecessary laboratory and radiological investigation. The long term outcomes of SPC are unknown at this time due to lack of long term longitudinal studies in these patient population. SPC within the lungs are a common finding on chest CT in children with DS. They are most commonly located in the anteromedial region of the lung. The etiology of the cysts remains unclear but it has been hypothesized that they are secondary to lung hypoplasia, a known feature of DS. It is not known whether SPCs resolve as the DS child grows and their lungs mature.

## References

[1] McDowell K. M., Craven D. I. (2011). Pulmonary complications of down syndrome during childhood. *Journal of Pediatrics*.

[2] Joshi V. V., Kasznica J., Ali Khan M. A., Amato J. J., Levine O. R. (1986). Cystic lung disease in down's syndrome: a report of two cases. *Fetal and Pediatric Pathology*.

[3] Biko D. M., Schwartz M., Anupindi S. A., Altes T. A. (2008). Subpleural lung cysts in Down syndrome: prevalence and association with coexisting diagnoses. *Pediatric Radiology*.

[4] Gonzalez O. R., Gomez I. G., Recalde A. L., Landing B. H. (1991). Postnatal development of the cystic lung lesion of down syndrome: suggestion that the cause is reduced formation of peripheral air spaces. *Pediatric Pathology*.

[5] Cooney T. P., Thurlbeck W. M. (1982). Pulmonary hypoplasia in Down's syndrome. *The New England Journal of Medicine*.

[6] Lytrivi I., Reingold S., Ramaswamy P. (2008). Neonatal left pulmonary artery occlusion and postinfarction cysts of the left lung: cause and effect?. *Pediatric Cardiology*.

[7] Gyves-Ray K., Kirchner S., Stein S., Heller R., Hernanz-Schulman M. (1994). Cystic lung disease in Down syndrome. *Pediatric Radiology*.

[8] Hoepker A., Seear M., Petrocheilou A. (2008). Wilson-Mikity syndrome: updated diagnostic criteria based on nine cases and a review of the literature. *Pediatric Pulmonology*.

[9] Griscom N. T., Wheeler W. B., Sweezey N. B., Kim Y. C., Lindsey J. C., Wohl M. E. B. (1989). Bronchopulmonary dysplasia: radiographic appearance in middle childhood. *Radiology*.

[10] Adams E. W., Harrison M. C., Counsell S. J. (2004). Increased lung water and tissue damage in bronchopulmonary dysplasia. *Journal of Pediatrics*.

[11] Oppenheim C., Mamou-Mani T., Sayegh N., De Blic J., Scheinmann P., Lallemand D. (1994). Bronchopulmonary dysplasia: value of CT in identifying pulmonary sequelae. *American Journal of Roentgenology*.

[12] Stocker J. T. (2009). Cystic lung disease in infants and children. *Fetal and Pediatric Pathology*.

[13] Esther C. R., Barker P. M. (2004). Pulmonary lymphangiectasia: diagnosis and clinical course. *Pediatric Pulmonology*.

[14] Verlaat C. W. M., Peters H. M., Semmekrot B. A., Wiersma-van Tilburg J. M. (1994). Congenital pulmonary lymphangiectasis presenting as a unilateral hyperlucent lung. *European Journal of Pediatrics*.

